# Evaluation of the relationship between the Desire to Avoid Pregnancy Scale and contraceptive use among women living with HIV in western Kenya

**DOI:** 10.3389/fgwh.2026.1803431

**Published:** 2026-08-28

**Authors:** Briend Donanty Kilone, Surya Sruthi Bhamidipalli, Caroline Kerich, Sammy Changwony, John Humphrey, Mercy Maina, Julie Thorne, Shukri A. Hassan, Mehar Maju, Kara Wools-Kaloustian, Beatrice Jakait, Rena C. Patel, Caitlin Bernard

**Affiliations:** 1 Department of Epidemiology, University of Alabama School of Public Health, Birmingham, AL, United States; 2 Department of Biostatistics, Indiana University School of Medicine, Indianapolis, IN, United States; 3 Academic Model Providing Access to Healthcare (AMPATH) Program, Eldoret, Kenya; 4 Department of Medicine, Indiana University School of Medicine, Indianapolis, IN, United States; 5 Moi Teaching & Referral Hospital (MTRH) - Pharmacy/AMPATH, Eldoret, Kenya; 6 Department of Obstetrics and Gynecology, University of Toronto, Toronto, ON, Canada; 7 Division of Infectious Diseases, Department of Medicine, Heersink School of Medicine, University of Alabama School of Medicine, Birmingham, AL, United States; 8 Department of Obstetrics and Gynecology, Indiana University School of Medicine, Indianapolis, IN, United States

**Keywords:** contraception [*methods], Desire to Avoid Pregnancy (DAP) scale, Kenya, pregnancy preferences, women living with HIV (WLHIV)

## Abstract

**Background:**

Unintended pregnancies place women living with HIV (WLHIV) at risk for maternal and neonatal adverse outcomes. Counseling around pregnancy preferences is important but complex, and there is insufficient evidence around best practices to assess and respond to reproductive health needs.

**Objective:**

This study aimed to compare responses to the Desire to Avoid Pregnancy (DAP) scale to assess pregnancy preferences, with the dichotomous traditional question “Are you trying to become pregnant within the next one year?” and how these relate to current contraceptive use.

**Methods:**

The study analyzed cross-sectional telephone interview data from the parent cohort study *Chaguo Langu* among WLHIV in western Kenya enrolled in HIV care in 2017–2020. Women underwent interviews about reproductive health and HIV care, including the DAP scale, the traditional question, and current contraceptive use. Multivariate logistic regression models were used to assess associations between DAP score, response to the traditional question, and contraceptive use, adjusting for age, marital status, and HIV treatment regimen.

**Results:**

Among 1226 participants, the median DAP score was 2.94 [IQR: 1.93–3.14] and was higher among those reporting not trying to become pregnant compared to those who were trying (3.0 vs. 0.9, *p* < 0.01), though the distribution of scores overlapped. In multivariable logistic regression, participants with a higher DAP score were more likely to use a contraceptive method.

**Conclusions:**

Among WLHIV, the DAP score captured a range of pregnancy preferences that correlated with the responses to the traditional dichotomous question and, less so, to contraceptive use. Further adaptations and testing would be needed to conclude whether DAP would be a better tool for assessment of nuances in pregnancy desires among WLHIV, including in Kenya, than the traditional dichotomous question.

## Introduction

Women make up the majority of people living with HIV, with an estimated 17.8 million women living with HIV (WLHIV) worldwide (1, 2). Over two-thirds of people living with HIV live in sub-Saharan Africa and Kenya is one of the countries most affected by HIV, with about 3% of Kenya's population (1.6 million people) living with HIV (3). Similarly, unintended pregnancies are a concern worldwide, and uniquely so for WLHIV. WLHIV experience up to ten times the maternal mortality rate of uninfected women (4). Fetal and infant effects of unintended pregnancy include the risk of vertical HIV transmission, with life-long consequences. In Kenya, about half of pregnancies in WLHIV are unintended (3), often related to the non-use or use of less effective contraceptive methods, such as condoms (5). At the same time, advances in antiretroviral therapy (ART) options and pre-exposure prophylaxis for HIV negative partners have increased the ability to safely plan pregnancies, resulting in increasing pregnancy desires for WLHIV (6). Therefore, it is more important than ever to truly understand women's pregnancy intentions in order to provide appropriate counseling and care for them to achieve their reproductive goals (6).

Over the past decade, advances in the treatment of HIV have prompted important discussions about how WLHIV are screened for pregnancy intentions and what counseling and resources are provided based on their responses (7). One such advance was the transition to dolutegravir (DTG)-containing ART as first-line ART due to its superior efficacy and tolerability, and its roll-out began in Kenya in 2017. However, the following year evidence emerged about a possible association between periconception DTG exposure in WLHIV and neural tube defects in their infants (8). The World Health Organization (WHO), and subsequently national health ministries including the Kenyan Ministry of Health (MoH), issued recommendations to assess women's pregnancy intentions and limit use of DTG containing ART to women using consistent, reliable contraceptive (9, 10). Even though this recommendation was later retracted with improved evidence of DTG safety in pregnancy (11) concerns about the use of DTG in women who may become pregnant have persisted and affected the uptake of DTG for WLHIV of reproductive potential (12).

Frequently, women are asked about their pregnancy intentions using the dichotomous traditional question, “Are you trying to become pregnant within the next one year?” Universal clinical implementation of this method of assessing pregnancy intentions, similar to the “One Key Question®”, has shown mixed results, with some potential improvement in patient satisfaction in primary care practices, but not clearly resulting in increased provision of reproductive counseling (13, 14). However, it remains the only previously implemented pregnancy intention assessment tool (15). Yet, women's intentions, preferences, and feelings around pregnancy and childbearing are often more complex and cannot always be captured by this dichotomous question, particularly when many women experience ambivalence or indifference to pregnancy (16). Thus, a critical gap exists in how best to capture pregnancy preferences in efficient but nuanced ways.

The Desire to Avoid Pregnancy (DAP) scale is meant to provide a more comprehensive approach to understanding pregnancy preferences in the research context and, therefore, may be adapted for use to help guide more comprehensive clinical counseling (17). The DAP scale encompasses a wide range of factors that women may consider related to their desire to become pregnant or avoid a pregnancy, including cognitive self-evaluation of preferences, affective feelings, and anticipated practical consequences of becoming pregnant within three months or having a baby within one year (17). It was first developed and validated for use in the United States in 2019 (17) and has since been adapted and validated in the United Kingdom, Botswana, India, and Brazil, and is underway in Uganda (15, 18, 19).

In Kenya, the uptake of contraception has increased in the last decade despite persisting gaps in provision (20). However, a study involving Kenyan women who desired to avoid pregnancy found that the use of contraceptive methods among WLHIV in Kenya was still suboptimal (21). Condoms, which are among the least effective contraceptive methods, continue to be among the most commonly used methods among WLHIV, potentially due to their promotion for dual use for HIV/sexually transmitted infections and pregnancy prevention (22). These findings highlight the need for increased effort towards improving reproductive and contraceptive counseling. This is particularly true for WLHIV due to the unique barriers and challenges they face related to the management of HIV, as became evident during the DTG and neural tube issue. Thus, methods to better capture WLHIV's pregnancy preferences are needed, coupled with optimization of contraception counseling, to improve overall reproductive health outcomes for WLHIV. While the DAP scale has the potential to capture pregnancy preferences comprehensively, there are significant differences in the sociodemographic contexts of Kenya and the United States; therefore, further research on the validity and utility of the DAP scale is needed for WLHIV in Kenya. Thus, the objective of this study was to pilot test the DAP scale among WLHIV in Kenya to assess its overall performance and associations with contraceptive use.

## Methods

The parent study for this data is titled *Chaguo Langu – My Choice* and some of the study's results regarding DTG uptake and contraceptive use, the impact of COVID-19 on contraceptive access, and qualitative data from WLHIV about the concern for neural tube defects with DTG have been published elsewhere (7, 9, 23, 24). The clinical data available in the Academic Model Providing Access to Healthcare (AMPATH) program electronic medical record (EMR) collected by clinicians during routine care (25) was used for this study. AMPATH works within the Kenyan MoH facilities to provide HIV services based on national guidelines to more than 150,000 patients and participates in the International Epidemiology Databases to Evaluate AIDS consortium (IeDEA). The study was approved by the Institutional Research and Ethics Committee at Moi University and Moi Teaching and Referral Hospital in Kenya and the University of Washington Human Subjects Division in the United States. Based on the use of de-identified data, the study was deemed exempt from IRB review at Indiana University.

### EMR cohort selection

The *Chaguo Langu* study was a cohort study that included prospectively collected EMR data for WLHIV enrolled in HIV care at public-sector health facilities supported by the AMPATH and exposed to DTG for ART from 2017 to 2020. The parent study included WLHIV if they were: enrolled in HIV care at an AMPATH-supported facility and initiated DTG-containing ART on or after 1 October 2017, 15–49 years of age (inclusive) at the time of DTG initiation, and initiated DTG ≥12 months prior to February 6th, 2020 (database closure). The cohort was comprised of women 15–49 years of age in accordance with the WHO definition of reproductive potential (26).

### Telephone surveys

As part of the *Chaguo Langu* study, a subset of patients identified through data from the EMR were contacted to participate in telephone surveys to confirm previously collected data. For the telephone surveys, a subset of the above EMR cohort was sampled based on a combination of age, DTG initiation status, and pregnancy status. The survey included information about their HIV treatment, as well as questions about their reproductive health and HIV care decision-making, including the verbally administered DAP scale, the traditional question about pregnancy intentions in the next year, and current contraceptive method use. A further subset of telephone survey participants also underwent in-depth interviews, sampled based on whether they continued with DTG treatment, switched off of DTG, or became pregnant while on DTG. Results from the in-depth interviews have been published elsewhere (24). The full telephone survey can be found in the Supplementary Materials. All participants provided verbal consent and were advised that they did not have to answer any questions they did not want to. Trained research assistants collected data by verbal surveys conducted by telephone and data were input directly into RedCap. This analysis of the telephone survey data is further restricted to participants who completed the DAP scale portion of the telephone survey and did not report previous sterilization or hysterectomy.

### Instrument

**Figure 1:**
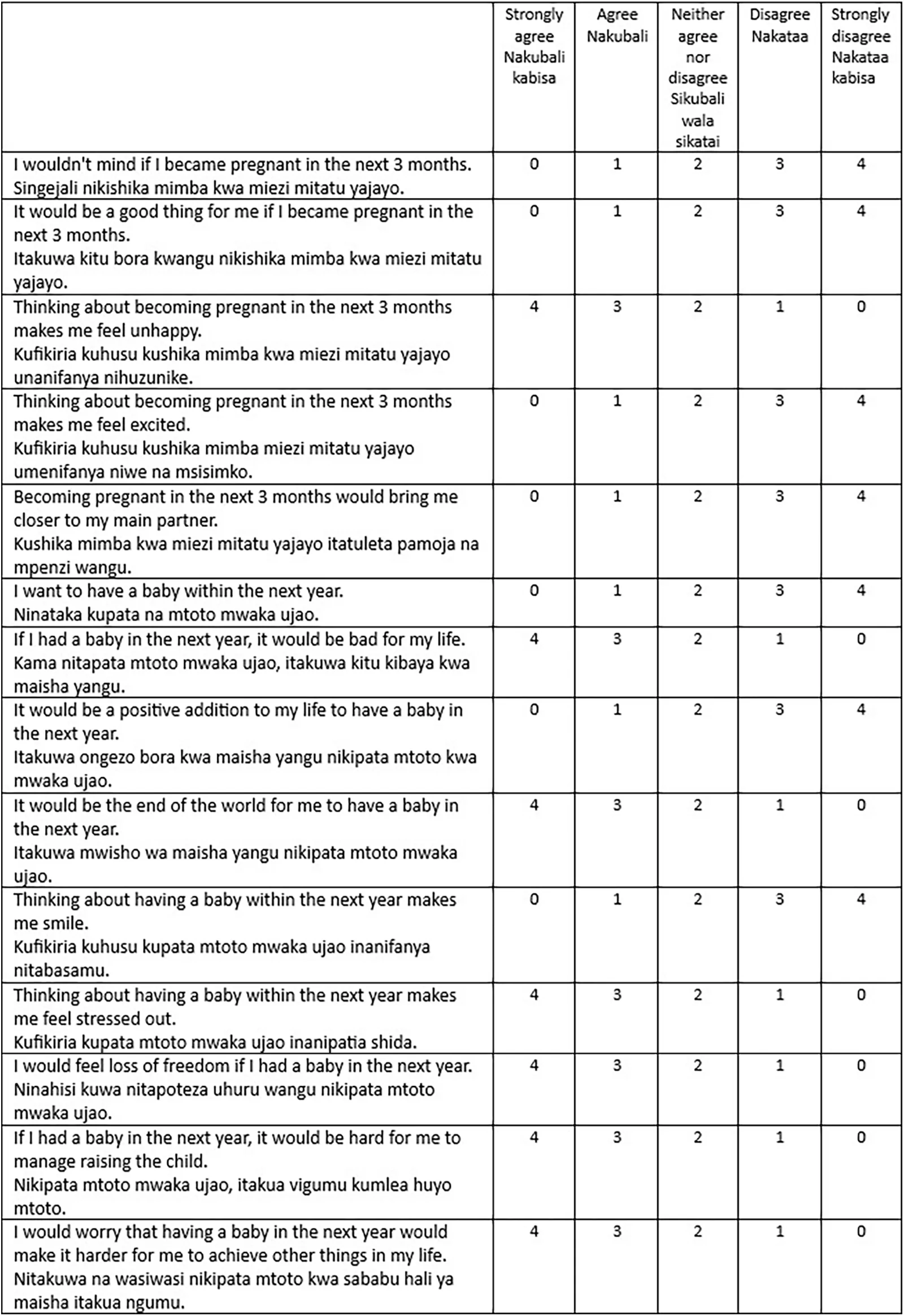
The Desire to Avoid Pregnancy (DAP) scale in English with Kiswahili translation with scoring system. Item scoring is additive and then divided by 14 to determine the final scale score from 0 to 4.

The methods for creating the DAP scale are detailed elsewhere (17). Briefly, the 14-question DAP scale was created using preference construction theory, which suggests that individuals often do not have clear preferences, especially those involving complex or context-specific choices. The scale was developed through cognitive interviews and psychometric analysis of field-testing data from the United States among women aged 15–45 years, who were sexually active in the last year, not sterilized, could speak and read English or Spanish, and not currently pregnant. The scale was specifically created to address the desire to *avoid* pregnancy, with a higher score indicating a greater desire to avoid pregnancy (Figure 1). Women respond to each DAP item on an ordinal Likert with values varying from 0 to 4, with 4 corresponding to the highest desire to avoid pregnancy, depending on the positive or negative wording of the item. The final DAP scale score is the mean of scores for all 14 items. The DAP scale was verbally administered during the telephone survey, reflecting the participant's responses at that time point. It was then translated from the original English version to Kiswahili, the primary language spoken in the study area, and then back-translated into English to check for accuracy. This was performed by research team members who are dual English and Kiswahili speakers, readers, and writers, with consensus on translation built among multiple members of the study team. The DAP scale questionnaires were administered in either English or Kiswahili based on the participant's preferred language, and trained research assistants conducted interviews.

Other survey questions included the current standard of care pregnancy intention assessment question, “Are you trying to become pregnant in the next year?” (16). In this telephone survey, it was used as a categorical variable with two response options: “yes” for those who intended to become pregnant in the next year and “no” for those who did not intend to become pregnant within a one-year period.

### Outcomes

Current contraceptive method use was determined based on the questions “Are you using any family planning method now?” and specific contraceptive method types, including condoms, injectable contraception, contraceptive implants, intrauterine device (IUD), oral contraceptive pills, permanent surgical methods, barrier methods, lactational amenorrhea, and others. More effective contraceptive use was defined as injectables, implants, and IUDs (27, 28), and participants were excluded from the DAP scale survey questions and therefore this analysis if they were using permanent methods.

### Covariates

Sociodemographic characteristics such as age, marital status, number of living children, number of years in school completed for education status, and site of care were collected. HIV characteristics collected included WHO HIV stage, years on ART at the start of their DTG-containing ART use, and current ART regimen.

### Analysis methods

Sociodemographic variables and HIV/ART characteristics were summarized using frequencies and percentages for categorical variables and means, medians, and interquartile ranges [IQR] for continuous variables. The associations between the DAP scale (exposure) and outcomes were initially evaluated using correlation coefficients, and then represented the results using violin plots. Next, logistic regression was performed, with multiple imputations for the missing values in the covariates via iterative chained equations with 25 multiply imputed datasets, to determine the association between DAP score and current contraceptive use, which included any method and more effective methods only. Odd ratios (ORs) were generated with confidence intervals (CIs) for both unadjusted and adjusted models. Adjusted models included site, age, marital status, number of living children, WHO HIV stage, and current ART regimen. Separate models were conducted using DAP score as a continuous vs. categorical variable (<2 vs. ≥2) based on previous work with the DAP scale (29). To compare the performance of the DAP score and the traditional question in relation to contraceptive use, the same models were also conducted with the traditional question as the exposure. The analysis was conducted using SAS 9.4 (NC, Cary).

## Results

### Overall characteristics

**Figure 2:**
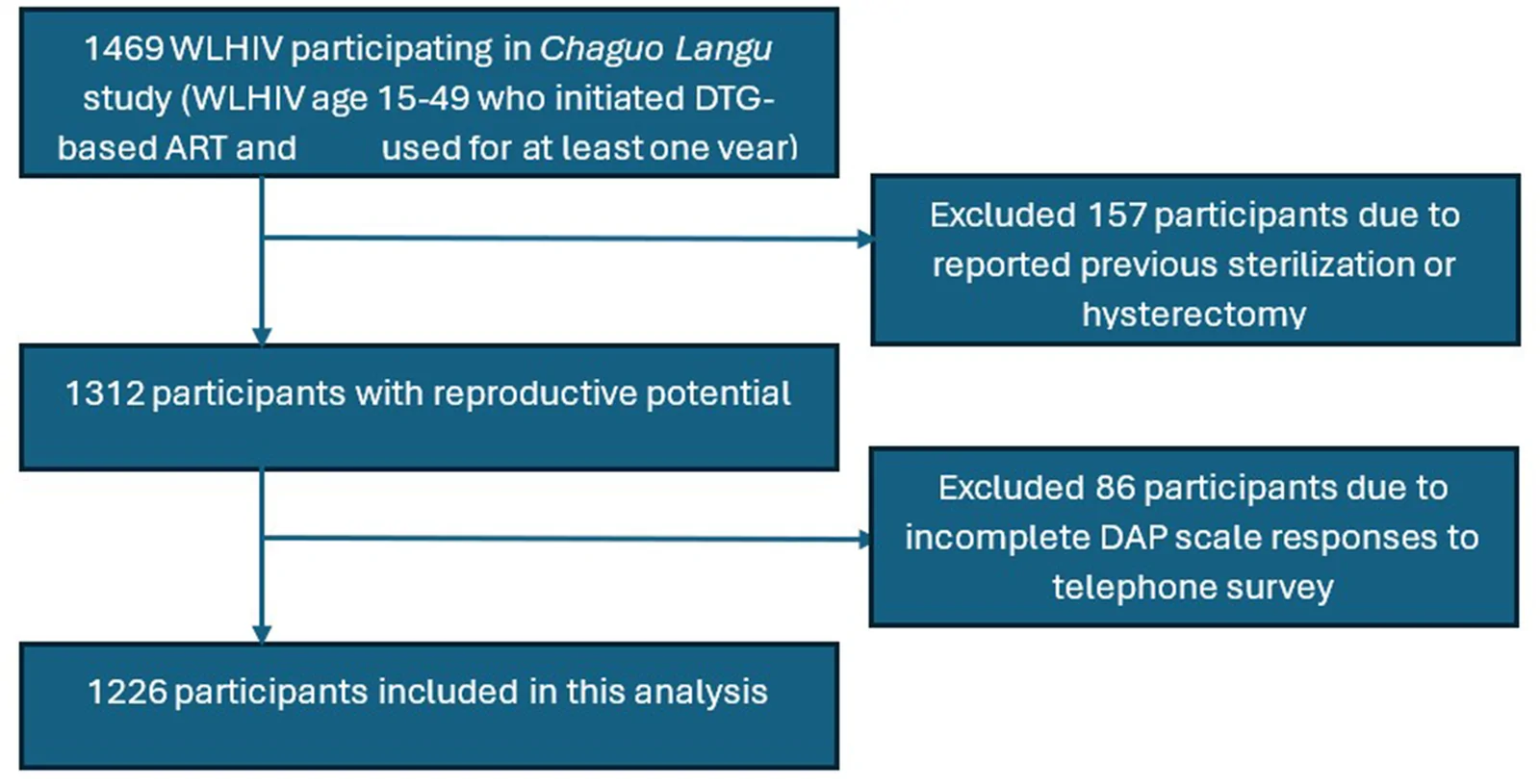
Participant selection flow chart.

**Figure 3:**
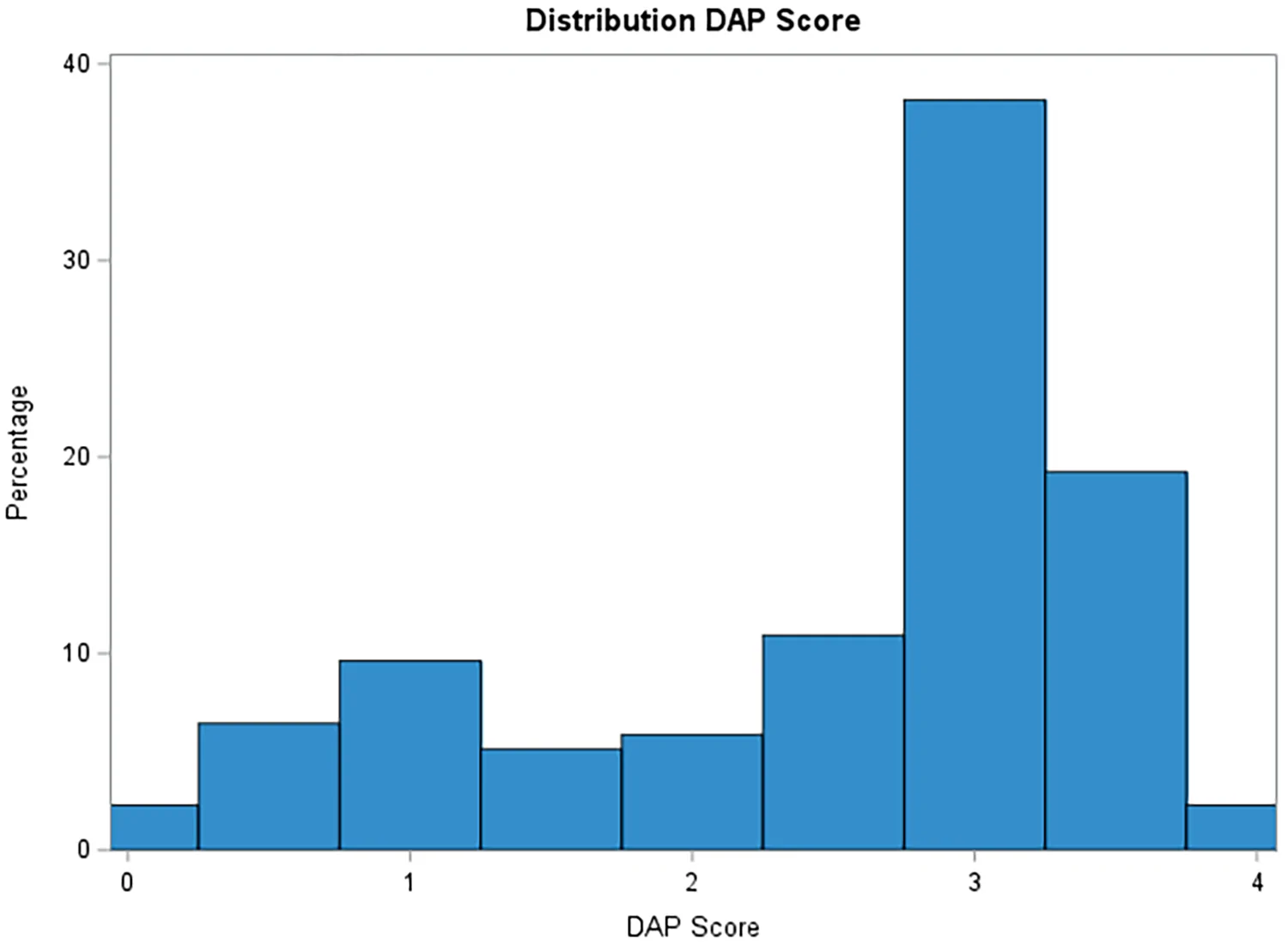
Distribution of Desire to Avoid Pregnancy Scale scores. Overall the mean DAP score was 2.51 with a range of 0–4 and the median score was 2.94 with an interquartile range of 1.93–3.14.

Of the 1,402 total participants undergoing the telephone survey, this analysis included data from 1,226 WLHIV (see Figure 2 for participant study flow including exclusions due to current pregnancy or previous sterilization or hysterectomy precluding future pregnancy). The women's median age was 38 years and more than half (54%) were either married or cohabiting (Table 1). The majority (90%) had at least one living child, and almost half (48%) had 3 or more children. More than half (61%) reported using any contraception and 40% were using a more effective method of contraception. The majority (89%) reported that they were not trying to get pregnant in the next year. Overall the mean DAP score was 2.51 and the median score was 2.94 with an interquartile range of 1.93–3.14 (Figure 3).

**Table 1:** Sociodemographic, HIV-related, and reproductive characteristics of *Chaguo Langu* participants included in this analysis (*N* = 1,226).

Sociodemographic characteristics	*n* (%) or Median (IQR)
Age (mean, in years) (*N* = 1,215)	38 (31–43)
Marital status	
Married/cohabiting	650 (53.9)
Single/Widowed	557 (46.1)
Missing	19
Number of living children	
0	120 (10.4)
1 or 2	476 (41.2)
3 or 4	409 (35.4)
>4	149 (12.9)
Missing	72
Number of years in school (in years) (*N* = 811)	8 (7–12)
Site of care	
Moi Teaching and Referral Hospital	298 (24.3)
Other	928 (75.7)
HIV/ART characteristics	
WHO HIV Stage	
Stage 1	606 (57.4)
Stage 2	197 (18.7)
Stage 3	205 (19.4)
Stage 4	48 (4.5)
Missing	170
Years of ART experience at the start of DTG use [*N* = 769]	2 (2–2)
Total years of ART use	
<1 year	0
1–5 years	761 (99)
>5 years	8 (1)
Missing	457
Current ART regimen	
Dolutegravir-based	676 (55.3)
Efavirenz-based	487 (39.8)
Nevirapine-based	26 (2.1)
Other	34 (2.8)
Missing	3
Current Contraceptive Method Use	
Are you using any family planning method now? (*n* = 1,226)	
Yes	751 (61.3)
No	475 (38.7)
Contraceptive method(s) (*n* = 751)	
Contraceptive implant	228 (30.4)
Condoms	227 (30.2)
Injectable contraception	191 (25.4)
Intrauterine contraceptive device	74 (9.8)
Oral contraceptive pills	29 (3.9)
Other	2 (0.3)
More effective methods^a^	493 (65.6)
Are you trying to get pregnant within the next year?	
No	1,075 (88.9)
Yes	133 (11.0)
Missing	18

WHO, World Health Organization; ART, antiretroviral therapy.

a Includes contraceptive implant, injectable contraception, and intrauterine contraceptive device.

### Comparison of DAP score to response to traditional pregnancy intentions question

**Figure 4:**
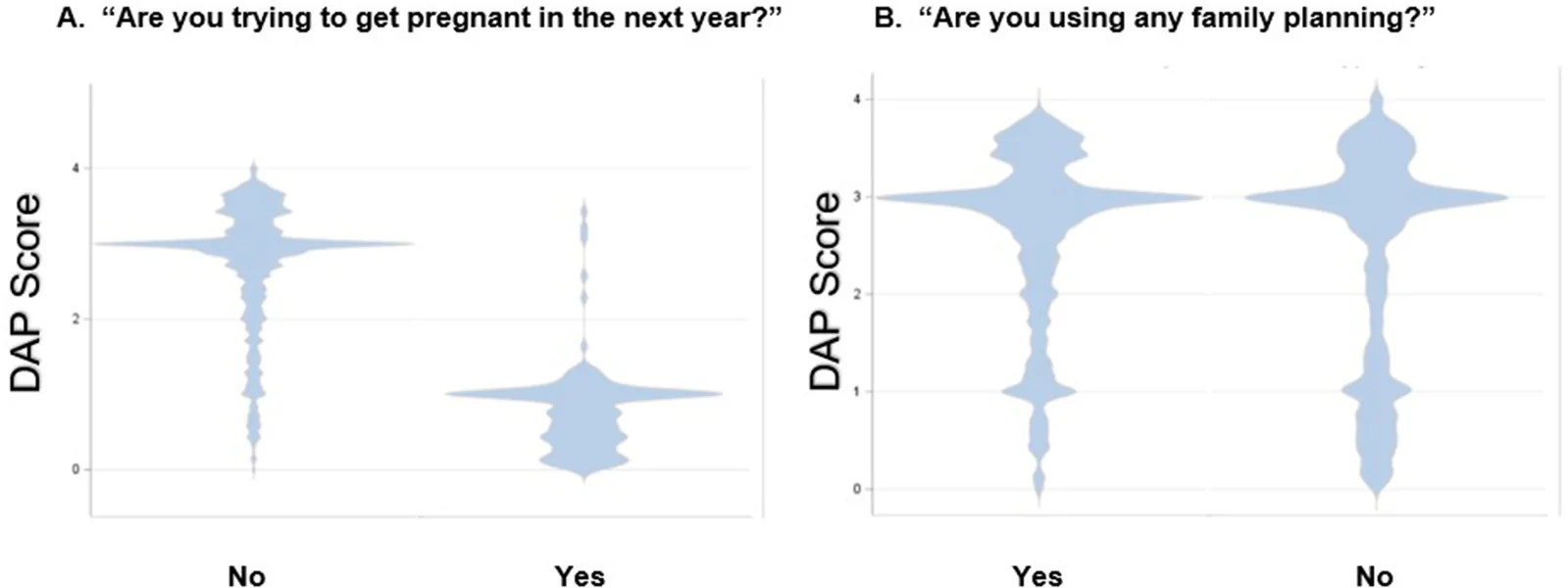
Violin plot representation of the correlations between Desire to Avoid Pregnancy (DAP) scale scores, “gold standard” pregnancy intentions question, and any contraceptive use (*N* = 1,208). **(A)** Correlation between DAP score and the “gold standard” question “Are you trying to get pregnant within the next year?” **(B)** Correlation between DAP score and any current contraceptive use.

The participants who responded to the traditional pregnancy intention question that they were not trying to get pregnant within the next one year had a higher median DAP score (3.00, IQR 2.43–3.21), correlating with a higher desire to avoid pregnancy, compared to those who reported they were trying to get pregnant within the next year (median score 0.86, IQR 0.43–1.00; Table 2). The violin plot distribution of the DAP scores visually demonstrates this relationship (Figure 4) but also shows a significant number of participants who report not trying to become pregnant have lower DAP scores. Fewer participants who report they are trying to get pregnant within the next year have higher DAP scores.

**Table 2:** Correlation of the Desire to Avoid Pregnancy (DAP) scale score with response to “gold standard” pregnancy intention screening question, current contraceptive use, and marital status.

Variable	Mean, Median DAP score (IQR)	*P*-Value*
Are you trying to get pregnant within the next year? (*n* = 1,208)		<0.01
No	2.72, 3.00 (2.43–3.21)	
Yes	0.81, 0.86 (0.43–1.00)	
Are you using any family planning method now? (*n* = 1,226)		0.03
Yes	2.60, 2.93 (2.14–3.14)	
No	2.37, 2.93 (1.29–3.14)	
Marital Status (*n* = 1,207)		<0.01
Married/cohabiting	2.46, 2.86 (1.86–3.00)	
Single/widowed	2.58, 3.00 (2.00–3.29)	

*P-Value obtained from Kruskal–Wallis test.

### Associations between DAP scores, traditional question response, and contraceptive use

When comparing pregnancy intentions and contraceptive use, significantly more women who responded to the traditional pregnancy intention question that they were not trying to get pregnant in the next year were using contraception (64.1% vs. 40.6%, *p* < 0.01, Table 3). However, more than 35% of participants who reported not trying to become pregnant were not using any method of contraception (Table 3). When comparing DAP score and contraceptive use, no difference was initially found between the median DAP score of women currently using any contraception (2.93, IQR 2.14–3.14) and those not using any contraception (2.93, IQR 1.29–3.14) [Figure 4B]. However, in multivariable logistic regression models, a significant relationship between DAP score and contraceptive use was found, including for both any method and more effective method use (Table 4). Those with a higher DAP score as a continuous variable were more likely to use any method of contraception (adjusted OR 1.22; 95% CI 1.07–1.39) and even more likely to use a more effective method (adjusted OR 1.44; 95% CI 1.25–1.65).

**Table 3:** Distribution of responses to the ‘gold standard’ pregnancy intention screening question by contraceptive use and marital status.

	Are you trying to get pregnant within the next year?		*P*-value*
	Yes	No	
Are you using any family planning method now? (*n* = 1,208)			<0.01
Yes	54 (40.6%)	689 (64.1%)	
No	79 (59.4%)	386 (35.9%)	
Marital Status (*n* = 1,189)			0.03
Married/cohabiting	81 (63.3%)	564 (53.2%)	
Single/Widowed	47 (36.7%)	497 (46.8%)	

*P-value obtained from a chi-square test.

**Table 4:** Logistic regression analysis comparing Desire to Avoid Pregnancy (DAP) scale score (measured as continuous and categorical variables) to contraceptive method use (measured as any method use and more effective method use).

Variable (*N* = 1,226)	Any contraceptive method use^b^		More effective contraceptive method use^c^	
	Unadjusted OR (CI)	Adjusted OR^a^ (CI)	Unadjusted OR (CI)	Adjusted OR (CI)
DAP Score (continuous)	1.24 (1.11–1.39)	1.22 (1.07–1.39)	1.52 (1.34–1.72)	1.44 (1.25–1.65)
DAP Score				
≥2	1.69 (1.30–2.19)	1.58 (1.19–2.11)	2.73 (2.04–3.66)	2.48 (1.80–3.41)
<2	Reference	Reference	Reference	Reference

Unadj. OR, unadjusted odds ratio; CI, confidence interval; Adj. OR, adjusted odds ratio.

a The adjusted model includes covariates such as site, age, marital status, number of living children, WHO HIV stage, and current ART regimen base.

b Any contraceptive method use includes contraceptive implants, injectables, intrauterine devices (IUDs), pills, condoms, and other.

c More effective contraceptive method use includes only contraceptive implants, injectables, and IUDs.

The DAP score was also explored as a categorical variable, which may be more helpful in a clinical context when determining the need for differential counseling. When the DAP score was used as a categorical variable, participants with a DAP score ≥2 compared to those with a DAP score <2 were more likely to use any method (adjusted OR 1.58; 95% CI 1.19–2.11) and much more likely to use a more effective method (adjusted OR 2.48; 95% CI 1.80–3.41). Full model results are included in Supplementary Tables S1–S4. In multivariable logistic regression, the traditional question was also correlated with both any contraception use (OR 2.48, 95% CI 1.67–3.68) and more effective method use (OR 5.37, 95% CI 3.03–9.52), see Supplementary Tables S5, S6).

## Discussion

Overall, the analysis showed that the DAP scale, translated into Kiswahili captures a range of pregnancy preferences among WLHIV in Kenya. When controlling for other variables, findings suggest that a higher desire to avoid pregnancy, as determined by a higher DAP score, is correlated with both contraceptive use and even more strongly correlated with more effective contraceptive method use. The dichotomous traditional question also appears to be correlated with both contraceptive use and more effective contraceptive method use. While the DAP score was designed to be used as a continuous variable, the DAP score was explored as a categorical variable, choosing the cut-off score of 2 based on previous work with the DAP scale (29). Future utility of the DAP scale in clinical settings will depend on making it clinically feasible and useful for providers and patients, thus creating score cut-offs to guide counseling may be useful. Conversely, this dichotomous cut-off may also risk losing the nuances that the DAP scale is specifically designed to help uncover. In comparison to the dichotomous traditional question used when assessing women's pregnancy intentions, the DAP scale offers a range of scores, allowing for better identification of ambivalent or unsure feelings about a potential pregnancy. This is particularly important for those participants who have seemingly conflicting responses, for example that they are not trying to get pregnant within the next year but have a low desire to avoid pregnancy as determined by a low DAP score. While it is difficult to interpret these findings without further qualitative analysis, this suggests a level of ambivalence or welcoming the idea of becoming pregnant or having a baby without clearly intending or trying to become pregnant. Women may also have conflicting preferences and actions related to unmeasured social pressures to become or prevent pregnancy in relation to their age, partner status, HIV status and other factors.

The results are consistent with prior studies' findings that there are nuances to women's reproductive preferences, and that the DAP scale may be superior to the dichotomous question because it can prospectively capture a range of preferences (29). Another US-based study that compared the DAP scale to the One Key Question® (“Would you like to become pregnant in the next year?”) found that DAP scale scores correlated with responses to the One Key Question® and contraceptive use (30). However, there was a range of pregnancy preferences as determined by the DAP score for each single response to the One Key Question®, reinforcing the importance of nuanced discussions that can be guided by having more information such as that provided by the DAP score.

Importantly, while the cohort's use of any method of contraceptive use at 61% was similar to other studies of Kenyan women (31) and higher DAP score was predictive of any and more effective method use, a significant number of women with higher DAP scores were not using a method of contraception. While two-thirds of those using contraception were using more effective methods, another one-third were using condoms as their primary form of contraception, which are less effective at pregnancy prevention. These findings underscore the need for improved interventions to educate and improve access to effective contraceptive methods that better match their pregnancy preferences. Further research is needed to better understand the potentially complex factors influencing women who report a strong desire to avoid pregnancy but are not using more effective forms of contraception and interventions for individualized counseling for reproductive health decision-making. Given that the median age of the cohort is 38 years, there may be perceptions of low or no risk of becoming pregnant with advanced reproductive age; directed counseling about the potential risk of pregnancy even at later ages may be needed. Counseling may also need to address commonly reported barriers such as fear of side effects and access to limited methods based on availability or health care provider skills, as well as potential unique barriers faced by WLHIV (32). Conversely, 40% of women who reported trying to become pregnant in the next year were using contraception, potentially indicating they were still in the process of delaying pregnancy until the right time or potentially other unknown factors related to their seemingly inconsistent preferences vs. practices.

Pregnancy, motherhood, and children carry significant cultural and social meanings, and also can pose significant socioeconomic and health challenges (32). As concerns around periconception dolutegravir use and potential risk of neural tube defects uncovered, effective tools to ascertain potentially complicated pregnancy preferences and guide reproductive counseling and care for WLHIV are urgently needed. The nuances around pregnancy preferences picked up with the DAP scale may help guide further counseling about the full spectrum of family planning, including preconception counseling. This underemphasized area of counseling is important to optimizing HIV care to prevent both partner and vertical transmission of HIV, as well as implementing other evidence-based interventions like folic acid supplementation. Similarly, the findings that a higher DAP score correlates well with both use of any contraceptive method and more effective methods support the potential utility of the DAP scale for guiding contraceptive counseling for WLHIV in Kenya. The DAP scale is a promising tool for efficient but nuanced discussions of pregnancy preferences and reproductive goals.

Ultimately, the DAP scale as a clinical tool must be coupled with counseling and interventions that optimize full-spectrum reproductive health, including supportive preconception care to effective contraceptive counseling. In the context of HIV, preconception counseling includes optimizing treatment for HIV and other chronic diseases; options for prevention of HIV transmission to serodiscordant partners, such as pre-exposure prophylaxis; folic acid, iron, and other prenatal vitamin supplementation; and discussions about pregnancy, delivery, and postpartum care, including birthing and breastfeeding plans. Similarly, counseling about pregnancy prevention would include discussions about methods of contraception that align with the patients' needs and future reproductive goals, as well as counseling around potential interactions of ART with specific contraceptive methods, such as known interactions between efavirenz and contraceptive implants. Most importantly, education must be paired with improved access to desired contraceptive methods, including the availability of permanent methods and improving consistency of access to short-term methods, which has been shown to be a barrier to effective contraceptive use in many areas (33).

The study is not without limitations. First, the use of telephone survey creates a selection bias for those with access to this technology. The responsiveness to phone calls depends on multiple factors, including the availability and willingness of the respondent to receive a call or respond to questions, the availability of a device, and access to the network, which impact the distribution of respondents and representativeness of the target population. Second, the translation of the DAP scale into Kiswahili may not allow for appropriate sociocultural contextual understanding of participants, thus, the ongoing work includes a DAP scale adaptation process, which may give better understanding and insight into this population. Similarly, the use of the traditional question may not have adequately captured those who might have responded with a third category such as “I don’t know” or “unsure”. While this is one argument for the use of a more well-rounded scale, such as the DAP scale, omitting this third choice may have missed some useful information. Third, the cohort of WLHIV older, has higher parity, and therefore likely an overall higher desire to avoid pregnancy than other populations, which may limit generalizability to younger, less parous populations. Nonetheless, the study cohort represents the aging phenomenon being observed, increasing in low- and middle-income countries within the HIV epidemic (34). Lastly, the study lacks data about the counseling that may have occurred between health providers and participants about their contraceptive options, to fully link the connections between pregnancy intentions or preferences and contraceptive use, particularly as WLHIV balance complex risks and benefits in their healthcare. Future studies should explore how eliciting pregnancy intentions or preferences through validated instruments can help guide patient-centered counseling and shared decision-making to ultimately empower women to achieve their reproductive goals. This is particularly important in the context of an already overburdened healthcare system caring for a population with limited health literacy and access to needed resources.

## Conclusion

Women's pregnancy preferences and reproductive health needs are more nuanced than what can be captured in the dichotomous question “Are you trying to become pregnant within the next year?” The DAP scale could be a promising tool to identify those nuances, including among WLHIV in Kenya. However, it showed a limited association with contraceptive method use. Studies that address limitations related to selection bias, socio-cultural influences, and to understand the preference for its use among health care providers and WLHIV could guide decisions about its usability and inform improvements to population-tailored versions for acceptability in clinical settings. A better understanding of the varied pregnancy preferences among WLHIV can help guide providers, health systems, and policymakers to improve the individualized counseling provided and access to reproductive healthcare to achieve individual reproductive goals.

## Data Availability

The data analyzed in this study is subject to the following licenses/restrictions: These are publicly available data and have no restrictions. The datasets are available upon request. Requests to access these datasets should be directed to Caitlin Bernard, caitlinb@iu.edu.
