# Supplementary material for: Evaluation of the relationship between the Desire to Avoid Pregnancy Scale and contraceptive use among women living with HIV in western Kenya
Source: Front Glob Womens Health. 2026 Aug 28;7:1803431. doi: 10.3389/fgwh.2026.1803431 (PMC13561986; doi:10.3389/fgwh.2026.1803431)
Supplement: Supplementary file 1 [file Table5.docx]

**Table 5: Logistic regression with outcome defined as “Yes” to “Are you using any family planning method now?”**

**which includes all the methods (implants, injectables, IUDs, pills, condoms, other) and gold standard question as**

**predictor with multiple imputation for the missing values.**

| **Variable (N = 1208)** | **Odds ratio** | **Confidence Intervals** |
| --- | --- | --- |
| **Site** |  |  |
| Other | 0.968 | 0.723-1.297 |
| Moi Teaching and Referral Hospital | **Reference** | |
| **Age** | 0.979 | 0.964-0.995 |
| **Marital Status** |  |  |
| Single/Widowed | 0.350 | 0.272-0.451 |
| Married/cohabiting | **Reference** | |
| **Number of living children** |  |  |
| 0 | 0.574 | 0.333-0.990 |
| 1 or 2 | 0.843 | 0.551-1.289 |
| 3 or 4 | 1.300 | 0.857-1.973 |
| >4 | **Reference** | |
| **WHO HIV stage** |  |  |
| Stage 1 | 1.144 | 0.646-2.026 |
| Stage 2 | 1.064 | 0.572-1.976 |
| Stage 3 | 0.982 | 0.532-1.812 |
| Stage 4 | **Reference** | |
| **New ART regimen base** |  |  |
| Dolutegravir based | 1.935 | 0.658-5.696 |
| Efavirenz based | 2.695 | 1.174-6.188 |
| Nevirapine based | 1.422 | 0.620-3.262 |
| Other | **Reference** | |
| **Are you trying to get pregnant now?** |  |  |
| **Yes** | 0.403 | 0.272-0.599 |
| **No** | **Reference** | |

**Table 6: Logistic regression with outcome defined as “Yes” to “Are you using any family planning method now?”**

**which includes only more effective methods (implants, injectables, IUDs) and gold standard question as predictor**

**with multiple imputation for the missing values.**

| **Variable (N = 1208)** | **Odds ratio** | **Confidence Intervals** |
| --- | --- | --- |
| **Site** |  |  |
| Other | 1.045 | 0.777-1.407 |
| Moi Teaching and Referral Hospital | **Reference** | |
| **Age** | 0.963 | 0.777-1.407 |
| **Marital Status** |  |  |
| Single/Widowed | 0.488 | 0.377-0.632 |
| Married/cohabiting | **Reference** | |
| **Number of living children** |  |  |
| 0 | 0.267 | 0.148-0.483 |
| 1 or 2 | 0.445 | 0.291-0.681 |
| 3 or 4 | 0.795 | 0.531-1.188 |
| >4 | **Reference** | |
| **WHO HIV stage** |  |  |
| Stage 1 | 0.935 | 0.513-1.701 |
| Stage 2 | 0.925 | 0.483-1.772 |
| Stage 3 | 0.793 | 0.418-1.507 |
| Stage 4 | **Reference** | |
| **New ART regimen base** |  |  |
| Dolutegravir based | 1.799 | 0.604-5.362 |
| Efavirenz based | 1.843 | 0.799-4.250 |
| Nevirapine based | 0.728 | 0.314-1.690 |
| Other | **Reference** | |
| **Are you trying to get pregnant now?** |  |  |
| **Yes** | 0.186 | 0.105-0.330 |
| **No** | **Reference** | |
